# Executive functioning, behavioural, emotional, and cognitive difficulties in school-aged children prenatally exposed to methadone

**DOI:** 10.3389/fped.2023.1118634

**Published:** 2023-04-18

**Authors:** Katherine M. Spowart, Kasey Reilly, Helen Mactier, Ruth Hamilton

**Affiliations:** ^1^ Specialist Children’s Services, NHS Greater Glasgow and Clyde, Glasgow, United Kingdom; ^2^ Princess Royal Maternity, NHS Greater Glasgow and Clyde, Glasgow, United Kingdom; ^3^ College of Medical, Veterinary and Life Sciences, University of Glasgow, Glasgow, United Kingdom; ^4^ Department of Clinical Physics and Bioengineering, Royal Hospital for Children, NHS Greater Glasgow and Clyde, Glasgow, United Kingdom

**Keywords:** prenatal methadone exposure, cognition, behaviour, long-term outcomes, prenatal tobacco exposure

## Abstract

**Aim:**

The aim of this study was to examine executive function and emotional and behavioural difficulties of children aged between 8 and 10 years who had been prenatally exposed to methadone, compared to non-exposed peers.

**Methods:**

Prospective study: third follow-up of an original cohort of 153 children born to methadone-maintained opioid-dependent mothers 2008–2010: previous investigations were at 1–3 days and at 6–7 months of age. Carers completed the Strength and Difficulties Questionnaire (SDQ) and the Behaviour Rating Inventory of Executive Function, Second Edition (BRIEF®2). Results were compared between exposed and non-exposed groups.

**Results:**

Carers of 33 of 144 traceable children completed the measures. SDQ responses showed no group differences on subscales of emotional symptoms, conduct problems, or peer relationship problems. A marginally higher proportion of exposed children had a high or very high hyperactivity subscale score. Exposed children scored significantly higher on BRIEF®2 behavioural, emotional, and cognitive regulation indices, and on the global executive composite. After controlling for potentially confounding higher reported maternal tobacco use in the exposed group *via* regression modelling, the effect of methadone exposure reduced.

**Interpretation:**

This study supports evidence that methadone exposure *in utero* is associated with adverse neurodevelopmental outcomes in childhood. Challenges in studying this population include difficulties with long-term follow-up and controlling for potentially confounding factors. Further investigation of the safety of methadone and other opioids in pregnancy must include consideration of maternal tobacco use.

## 1. Introduction

Opioid use in pregnancy has been widely reported to cause significant harm to children, evident both in the neonatal period and in later childhood (1, 2). In the neonatal period, children may suffer from neonatal abstinence syndrome/neonatal opioid withdrawal syndrome (NAS/NOWS) with prolonged hospital admission and/or maternal/infant separation and necessity for pharmaceutical treatment. The development of overt NAS/NOWS is not a prerequisite for adverse childhood outcome(s) (1), but the association of illicit opioid use with multiple obstetric complications may further impact longer-term outcomes (2, 3). Methadone is commonly used to manage opioid misuse in pregnancy with current guidelines stating that this practice is safe other than the risk of NAS/NOWS (4, 5). This advice does not concur with increasing evidence that prenatal opioid exposure is associated with increased risk of adverse neurodevelopmental outcomes, specifically impaired infant cognition and psychomotor performance, impaired early childhood internalising and externalising behaviour, and attention problems (6–9). Difficulties with executive functioning, vision (8), language, and regulation (9) are also reported.

Neurodevelopmental outcomes in later childhood and adolescence are less well understood although it would be predicted that lower cognitive performance in children aged over 2 years would carry a risk of longer-term difficulties (10). Indeed, in a longitudinal study of children prenatally exposed to opioids, group differences in cognition, attention, and behaviour had widened by 8 years of age (11, 12). Lower cognitive function compared to non-opioid-exposed controls has been described in 17- to 21-year-old youths although their performance was within normal limits (13). Unfortunately, studies in this field are limited methodologically because of the challenges of identifying polydrug and other licit [including tobacco (14) and alcohol] exposures, and the potentially confounding effects of these additional drug exposures as well as adverse pregnancy or neonatal illness, ill-health associated with poor socioeconomic status, and suboptimal childhood environment.

A prospective cohort study of infants born to methadone-maintained opioid-dependent (MMOD) mothers established polydrug exposures *via* both maternal and infant toxicology and recruited a comparison group matched for major confounding factors. The study was designed to investigate visual outcomes and found impaired neonatal visual evoked potentials (15) and significant visual problems at 6 months (16) and at 8–10 years. A subgroup of the cohort attended at 8–10 years for detailed visual investigation and both neurodevelopmental and behavioural enquiry. The aim of this study arm was to compare results of neurodevelopmental/behavioural carer-completed questionnaires at 8–10 years between exposed and comparison children.

## 2. Methods

### 2.1. Participants

Participants comprised 33 of 144 (98 exposed, 46 comparison) traceable children followed up at ages of 8–10 years. Exposed children (*n* = 21) were born to MMOD mothers and comparison (non-exposed) children (*n* = 12) were born contemporaneously (2008–2010) at the same maternity hospital. All were born after 36 weeks’ gestation; none had congenital ocular abnormality or significant neonatal illness. Prenatal drug exposure of infants born to MMOD mothers was established *via* maternal urine, infant urine and meconium, maternal casenote review, and confidential interview (15). A subgroup of comparison infants had meconium drug analysis. For both exposed and non-exposed newborns, a subset of meconium samples was analysed for prenatal alcohol exposure (PAE), with a fatty acid ethyl ester (FAEEs) concentration ≥10,000 ng/g considered to represent significant PAE (17). Comparison infants were matched at recruitment for completed week of gestation, birthweight (±250 g) and socioeconomic status [Carstairs deprivation index using postcode of residence (±1)] (18) and partially matched for maternal tobacco use. Selection bias was likely to be low due to the high consent rate (98%) at recruitment (19). Characteristics of the 33 children are detailed in Table 1. The 33 attending children closely matched the non-attending traceable children (*n* = 111) for birth characteristics and drug exposure.

**Table 1:** Characteristics of exposed and comparison children.

Maternal, birth, and neonatal characteristics	Exposed children (*n *= 21)	Comparison children (*n *= 12)	Difference (95% CI)	Test, *p*-value
Sex, (*n*) % male	(10) 48%	(3) 25%	23% (−12% to 48%)	FE, *p* = 0.18
Gestation, week^a^	39.4 (37.8–40.4)	39.9 (38.4–41.0)	−0.6 (−1.9 to 0.4)	MW, *p* = 0.28
Birthweight, g^b^	2,878 (448)	3,114 (550)	−236 (−626 to 154)	*t*-test, *p* = 0.22
Birth occipitofrontal head circumference, cm^b^	33.3 (1·8)	34.1 (1.7)	−0.8 (−2.1 to 0.5)	*t*-test, *p* = 0.23
Maternal tobacco use, (*n*) %	(21) 100%	(8) 67%	33% (3% to 33%)	*χ*^2^, *p* = 0.012
Reported cigarettes per day^a^	10 (10–15)	10 (0–10)	5 (0 to 10)	MW, *p* = 0.043
Known prenatal alcohol exposure, (*n*) %	6/15, 40%	1/5^c^, 20%	20% (−23% to 63%)	FE, *p* = 0.4
Maternal body mass index^a^	23 (21–25)	25 (22–33.75)	−3 (−8 to 0)	MW, *p* = 0.08
Maternal Carstairs deprivation index^a^	7 (4.5–7)	6.5 (5–7)	0 (−1 to 1)	MW, *p* = 0.9
NAS/NOWS, (*n*) %	(14) 67%	—	—	—
Drug exposure, (*n*) %				
Methadone	(21) 100%	0/7 tested		
Prescribed dose at delivery (mg/day)^a^	55 (40–80)	—		
Opiates	(19) 90%	0/7 tested		
Benzodiazepines	(16) 76%	1/7 tested		
Cannabis	(13) 62%	1/7 tested		
Amphetamine	(3) 14%	1/7 tested		
Cocaine	(4) 19%	0/7 tested		
Follow-up demographics and outcomes				
Age, years^b^	9.3 (0.7)	9.3 (0.7)	0.1 (−0.6 to 0.4)	*t*-test, *p* = 0.7
Birth mother deceased, (*n*) %	(3) 14%	(0) 0%	14% (−12% to 35%)	FE, *p* = 0.24
Adopted or in foster/kinship care, (*n*) %	(10) 48%	(3) 25%	23% (−12% to 48%)	FE, *p* = 0.18
Learning support at school, (*n*) %	(5/18) 28%	(1) 8%	19 (−12 to 44)	*χ*^2^, *p* = 0.20
Height, cm^b^	135 (7.1)	135 (5.4)	0.3 (−5 to 4)	*t*-test, *p* = 0.9
Weight, kg^b^	31.6 (7.5)	34.3 (8.1)	−2.6 (−8.6 to 3.3)	*t*-test, *p* = 0.37
Head circumference, cm^b^	53.0 (2.0)	53.3 (1.1)	−0.3 (−1.4 to 0.8)	*t*-test, *p* = 0.6
Visual outcome “fail,” (*n*) %	(14) 67%	(2) 17%	50% (8% to 71%)	FE, *p* = 0.01

CI, confidence interval; FE, Fisher’s exact test; MW, Mann–Whitney test; NAS/NOWS, neonatal abstinence syndrome/neonatal opioid withdrawal syndrome requiring pharmaceutical treatment; PAE, prenatal alcohol exposure.

^a^ Median (interquartile range).

^b^ Mean (standard deviation).

^c^ Due to data loss, PAE status is known only for one comparison child: denominator is unknown but assumed to be *n* = 5 based on neonatal data proportions.

**Figure 1:**
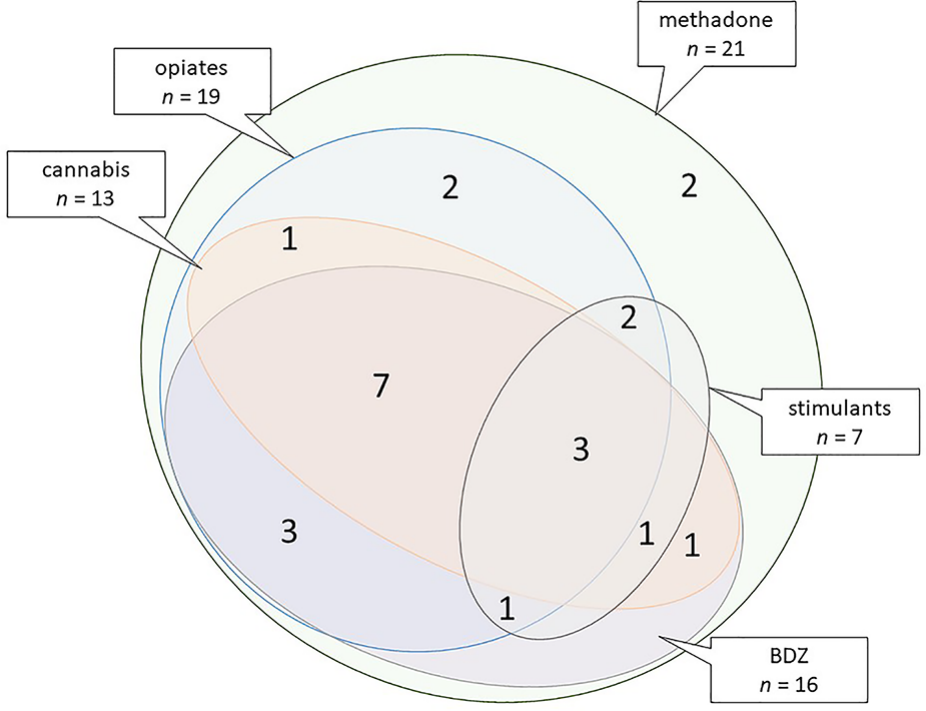
Euler diagram illustrating combinations of polydrug exposure based on combined exposure data for the 21 exposed children. Stimulants: cocaine and/or amphetamines. BDZ, benzodiazepines.

Exposed children were considered to have developed NAS/NOWS if they received pharmaceutical treatment according to the well-established hospital protocol. Oral morphine replacement was commenced (60 μg/kg × 6 per day) and weaned (usually by 10 μg/kg/day as symptoms diminished) when two consecutive 12-h scores >5 on a modified Lipsitz scale (20) were recorded in conjunction with poor feeding/weight gain. Second line phenobarbital was added when morphine treatment was unsuccessful (minority of babies). The median length of morphine treatment was 10 days; phenobarbital, if required, was generally weaned and discontinued by 6 weeks of age. All children had been prenatally exposed to methadone; most were exposed to additional drugs (Figure 1). Casenotes were reviewed for any attendance at hospital eye services, care arrangements (birth parent, adopted, or kinship or foster care), supported learning, or diagnosis of autistic spectrum disorder (ASD), attention deficit hyperactivity disorder (ADHD), and/or foetal alcohol spectrum disorder (FASD). Casenote review was performed by researchers masked to exposure status with limited bias potential as data collected were previously documented, objective findings.

### 2.2. Assessments

A paediatric research nurse documented care and education status, height, weight, and occipitofrontal head circumference (OFC). Detailed visual assessments were undertaken with predetermined fail criteria (acuity poorer than 0.2 logMAR not attributable to refractive error; any manifest strabismus or any nystagmus; inability to overcome any base-out prisms; or a Frisby stereothreshold >110 arcsec). A researcher applied two child behaviour questionnaires to accompanying adults, assisting where necessary and encouraging completion of all questions.

The Strengths and Difficulties Questionnaire (SDQ) (21) is a 25-item emotional and behavioural screening questionnaire with five subscales: emotional problems, conduct problems, hyperactivity and peer problems where high scores indicate more problems, and a prosocial subscale where high scores indicate fewer problems. Each item is scored on a Likert scale (not true = 0; somewhat true = 1, certainly true = 2) with possible subscales scores of 0–10. The total difficulties score is the sum of scores for the first four subscales (possible values 0–40). Scores are categorised as follows: close to average, slightly raised, high, or very high using “parent-completed” scores relative to a large UK reference population (22). The SDQ has high reliability, validity (23), and good concurrent validity (24, 25).

The Behaviour Rating Inventory of Executive Function, 2nd edition (BRIEF®2) (26) is a clinical rating scale of executive function comprising three regulation indices and a global executive composite (GEC). The behavioural regulation index (BRI) measures the child's ability to regulate and monitor their behaviour effectively and consists of “inhibit” and “self-monitor” scales. The emotion regulation index (ERI) measures the child's ability to regulate their emotional responses and to adjust to changes in environment, people, plans or demands, and consists of “shift” and “emotional” scales. The cognitive regulation index (CRI) measures the child's ability to control and manage cognitive processes and to problem solve, and consists of “initiation,” “working memory,” “planning,” “task-monitor,” and “organisation of materials” scales. The GEC is a summed score of all nine scales. The three indices and the GEC are expressed as *T*-scores. Scores are categorised as follows: mildly elevated (60–64 inclusive), potentially clinically elevated (65–69 inclusive), or clinically elevated (≥70). The test design incorporates checks for inconsistency (respondent answered similar items in an inconsistent manner), infrequency (respondent endorsed unlikely events), and negativity (respondent answered in an unusually negative manner) with criteria for exclusion.

Questionnaires were independently scored by two researchers (RH and KMS) and interpreted and analysed by researchers (KMS and KR) qualified to do so. Researchers were masked to exposure status to limit bias potential. Assessments took place at the paediatric Clinical Research Facility, Queen Elizabeth University Hospital campus, Glasgow, United Kingdom, between January 2018 and February 2020. Any child causing medical or social concern not already being addressed was notified to relevant services after discussion with their carer. Families were offered reimbursement of expenses and the child was given a £20 voucher. Written informed consent was given by the child's legal guardian; children gave written informed assent. The study was approved by West of Scotland Research Ethics Committee 3 (17/WS/0093).

### 2.3. Data analysis

SDQ scores were compared between exposed and comparison children using Mann–Whitney *U* tests; proportions of children with scores classified as “high/low” or “very high/low” were compared using Fisher’s exact test. BRIEF®2 scores were compared between exposed and comparison children using *t*-tests without the assumption of equal variance; proportions of children with scores classified as “potentially clinical elevated” or “clinically elevated” were compared using Fisher’s exact tests. To assess potential confounders, factors differing meaningfully between exposed and comparison children were treated as predictor variables in regression models of BRIEF®2 scores. SDQ total difficulties and BRIEF®2 GEC scores were compared using linear correlation to investigate whether an elevated score on one questionnaire was associated with an elevated score on the other. Findings were compared qualitatively with neurodevelopmental assessment undertaken at 6 months of age using the Griffiths Mental Development Scales (27). Findings for exposed children were compared for those who had and had not required treatment for NAS/NOWS (SDQ scores, Mann–Whitney *U* tests; BRIEF®2 scores, unpaired *t*-tests without assumption of equal variance). The relation between prescribed maternal methadone dose at delivery and questionnaire scores was investigated using scatter plots. Analyses were performed using SPSS® Statistics v24.0 (IBM Corp., Armonk, NY, United States), Minitab® v20.3 (Minitab LLC, PA, United States), and MedCalc® v20.014 (MedCalc Software Ltd, Ostend, Belgium).

## 3. Results

Exposed and comparison children did not differ in neonatal characteristics except for maternal tobacco use: all MMOD mothers but only two-thirds of comparison mothers smoked tobacco cigarettes (Table 1). Groups did not differ in childhood characteristics except for a greater fail rate on vision assessment for exposed children (Table 1). Individual child characteristics are shown in Table 2.

**Table 2:** Individual subject social, medical, and neurodevelopmental findings (case note review and history) and provision of learning support.

#	Exposed or comparison	Treated NAS	Sex	Age	Birth mother alive	Looked after status	Accompanying adult	Medical issues	Neurodevelopmental issues	Learning support at school	SDQ total difficulties score	BRIEF®2 GEC *T*-score	Griffiths general quotient
4	Exposed	Y	M	11.0	Yes	BP	Mother		ADHD (treated); ASD	No	30[Table-fn table-fn6]	86[Table-fn table-fn7]	97
6	Exposed	Y	F	10.6	Yes	BP	Father			No	8	56	88
7	Exposed	Y	M	9.2	Yes	BP	Mother			No	8	51	95
11	Exposed	Y	F	9.2	Yes	A	Mother			No	10	48	88[Table-fn table-fn8]
18	Exposed	Y	F	9.0	Yes	KC	Grandmother			No	15	54	82[Table-fn table-fn8]
21	Exposed	Y	F	9.1	Yes	BP	Mother			not known	7	56	99
25	Exposed		M	9.0	Yes	A	Mother			No	9	52	98
30	Exposed	Y	M	9.9	Yes	A	Mother	Asthma	ADHD	No	12	55	101
38	Exposed		F	10.2	Yes	BP	Mother			No	6	45	
43	Exposed	Y	F	10.7	Yes	KC	Grandmother	Headaches		No	15	61	97
44	Exposed		M	8.8	No	KC	Grandmother	Eczema	Being investigated for ADHD	not known	19[Table-fn table-fn6]	75[Table-fn table-fn7]	92
57	Exposed	Y	M	8.8	Yes	A/KC	Aunt = adoptive mother	Asthma	Being investigated for ASD	not known	28[Table-fn table-fn6]	80[Table-fn table-fn7]	104
74	Exposed	Y	F	9.5	Yes	BP	Father			No	17[Table-fn table-fn6]	58	93
77	exposed	Y	F	9.5	Yes	BP	Mother	Asthma, headaches		No	8	54	102
105	Exposed		M	8.9	Yes	BP	Mother	Hypermobility		No	14	72[Table-fn table-fn7]	93
109	Exposed	Y	F	8.8	No	BP	Father		ASD/ADHD concern	Yes	9	Inconsistent	99
112	Exposed	Y	F	9.1	No	KC	Grandfather			Yes	18[Table-fn table-fn6]	Negativity	100
141	Exposed		M	8.7	Yes	A	Mother	Dermatographia		No	6	47	92
143	Exposed	Y	F	8.7	Yes	BP	Mother			No	7	56	94
147	Exposed		M	8.8	Yes	BP	Father	Motor difficulties, language delay		Visual impairment teacher; child psychologist	30[Table-fn table-fn6]	81[Table-fn table-fn7]	97
153	Exposed		M	8.7	Yes	A	Mother			Reading help	19[Table-fn table-fn6]	63	
5	Comparison		M	10.5	Yes	BP	Mother			No	9	51	
55	Comparison		F	10.3	Yes	KC	Grandmother	Diabetes, coeliac disease, constipation		No capacity to write	16	Inconsistent	98
79	Comparison		F	9.7	Yes	BP	Mother	Constipation		No	2	42	102
80	Comparison		F	9.7	Yes	BP	Mother			No	4	47	96
100	Comparison		F	8.8	Yes	BP	Mother	Meningitis, hearing impairment		No	9	52	
115	Comparison		M	8.8	Yes	A/KC	Aunt = adoptive mother			No	7	54	
116	Comparison		F	9.4	Yes	BP	Father	Heart murmur from birth, constipation		No	18[Table-fn table-fn6]	Infrequency	100
125	Comparison		F	9.0	Yes	BP	Stepmother			No	7	45	114
130	Comparison		M	8.7	Yes	A/KC	Aunt = adoptive mother		ASD	No	18[Table-fn table-fn6]	Inconsistent	107
136	Comparison		F	8.6	Yes	BP	Mother	Chronic constipation		No	14	56	100
139	Comparison		F	8.9	Yes	BP	Mother	Hip dysplasia, ankle fractures		No	6	48	115
146	Comparison		F	8.7	Yes	BP	Mother	Enlarged bladder, constipation		No	14	Infrequency	102

NAS, neonatal abstinence syndrome; M, male; F, female; BP, birth parent; A, adopted; KC, kinship care; ADHD, attention deficit hyperactivity disorder; ASD, autistic spectrum disorder; BRIEF®2, Behaviour Rating Inventory of Executive Function, 2nd edition; GEC, global executive composite; SDQ, strengths and difficulties questionnaire.

* SDQ total difficulties score classified as “high” or “very high.”

** BRIEF®2 GEC score classified as “potentially clinically significant” or “clinically significant.”

*** Any Griffiths sub-quotient scored “low” or “very low.”

### 3.1. SDQ

All SDQ screening questionnaires (*n *= 33) were completed adequately. Details of accompanying adult who completed the SDQ (e.g., birth mother, grandparent, etc.) are given in Table 2. Exposed children and comparison children scored similarly on all subscales and total difficulties scores. Similar proportions of exposed and comparison children had “high” or “very high” scores in three subscales and in total difficulties. A marginally greater proportion of exposed children had “high” or “very high” scores on the hyperactivity subscale (more children with hyperactive behaviour, Table 3).

**Table 3:** SDQ results.

	Median score, exposed children (*n* = 21)	Median score, comparison children (*n* = 12)	95% CI of difference; Mann–Whitney *U*test *p-*value	Proportion of exposed children classified with “high/low” or “very high/low”	Proportion of comparison children classified with “high/low” or “very high/low”	95% CI of difference; Fisher’s exact test *p-*value
Emotional problems subscale	3	3	−1 to 3 *p* = 0.47	9/21	2/12	−7% to 50% *p* = 0.12
Conduct problems subscale	1	1	−1 to 2 *p* = 0.50	5/21	2/12	−24% to 32% *p* = 0.5
Hyperactivity subscale	5.0	4.5	−0 to 4 *p* = 0.13	6/21	0/12	0.2% to 50% *p* = 0.049
Peer relationships problems subscale	2.0	1.5	−1 to 2 *p* = 0.43	7/21	3/12	−24% to 35% *p* = 0.5
Total difficulties	12	9	−1 to 8 *p* = 0.20	7/21	2/12	−16% to 41% *p* = 0.4
Prosocial subscale	9	10	−2 to 0 *p* = 0.34	3/21	2/12	32% to −21% *p* = 0.6

CI, confidence interval; SDQ, Strength and Difficulties Questionnaire.

Total difficulties is the sum of scores from the first four subscales where a high score indicates more problems.

### 3.2. BRIEF®2

Six BRIEF®2 questionnaires were excluded from analysis (two exposed and four comparison children). Three were excluded for inconsistency (respondents: one birth father, one grandparent, and one adoptive mother), two for infrequency (respondents: one birth mother and one birth father). and one for negativity (respondent: grandparent). Data were, therefore, available for 19 exposed children and 8 comparison children. Exposed children scored significantly higher than comparison children on all three regulation indices (behavioural, emotional, and cognitive) and on their total score (GEC) (Table 4). Five of the 19 (26%) exposed children had a clinically elevated GEC. No comparison child had any clinically elevated or potentially clinically elevated index. Three exposed children (#004, #147, and #057) had all three indices clinically elevated, one child (#044) had clinically elevated BRI and CRI, and one child (#105) had clinically elevated ERI and potentially clinically elevated CRI (Tables 2, 4). Of these five children, one had a diagnosis of ADHD and ASD, one was being investigated for ADHD, one was being investigated for ASD, and one was known to have motor and speech difficulties. Statistically significant differences in the proportions of children with potentially clinically elevated and/or clinically elevated indices were not found: large confidence intervals (CIs) indicate a small sample size effect, with only eight comparison children contributing to BRIEF®2 data (Table 4). Regression modelling showed that the effect size (higher BRIEF®2 scores for exposed children) reduced for all indices and for GEC after controlling for maternal tobacco use: methadone exposure no longer predicted higher BRIEF®2 GEC scores after controlling for maternal tobacco use (Table 5). Controlling for maternal tobacco use changed BRI effect size most markedly and had a minimal effect on ERI (Table 5).

**Table 4:** BRIEF®2 results.

	Mean T-score, exposed children (*n* = 19)	Mean T-score, comparison children (*n* = 8)	95% CI of difference; Mann–Whitney *U* test *p-*value	Proportion of exposed children classified with “potentially” or “clinically significant”	Proportion of comparison children classified with “potentially” or “clinically significant”	95% CI of difference; Fisher exact test *p-*value
BRI	58	49	2 to 16 *p* = 0.015	4/19	0/8	−14% to 43% *p* = 0.22
ERI	61	49	5 to 18 *p* = 0.002	5/19	0/8	−9% to 49% *p* = 0.14
CRI	58	49	2.5 to 14 *p* = 0.007	5/19	0/8	−9% to 49% *p* = 0.14
GEC	61	49	4 to 18 *p* = 0.002	5/19	0/8	−9% to 49% *p* = 0.14

CI, confidence interval; BRIEF®2, Behaviour Rating Inventory of Executive Function, 2nd edition; BRI, behaviour regulation index; ERI, emotional regulation index; CRI, cognitive regulation index; GEC, global executive composite.

GEC is the sum of the three regulation indices where a high score indicates more problems.

**Table 5:** Regression parameters: association of methadone exposure with BRIEF®2 scores before (unadjusted) and after (adjusted) controlling for maternal tobacco use.

	Unadjusted effect size—score difference between exposed and comparison children (SE)	*p-*value	Adjusted effect size (SE)	Adjusted *p-*value
BRI	9.0 (4.1)	0.04	5.8 (4.8)	0.2
ERI	11.6 (4.7)	0.02	10.3 (5.7)	0.085
CRI	8.4 (3.7)	0.03	5.7 (4.4)	0.2
GEC	11.2 (4.5)	0.02	8.3 (5.4)	0.14

BRIEF®2, Behaviour Rating Inventory of Executive Function, 2nd edition; BRI, behaviour regulation index; ERI, emotional regulation index; CRI, cognitive regulation index; GEC, global executive composite.

### 3.3. SDQ and BRIEF®2 concordance

**Figure 2:**
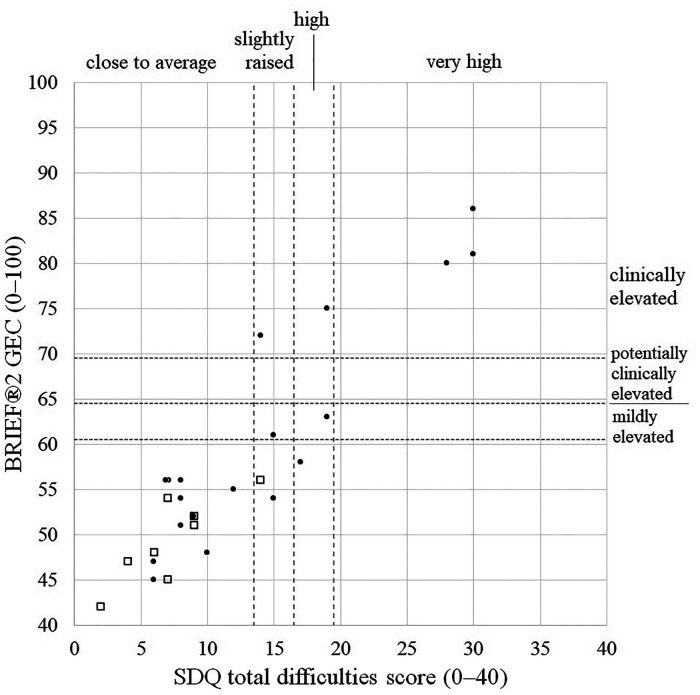
Scatterplot of BRIEF®2 GEC scores vs. SDQ total difficulties scores illustrating degree of concordance. Closed circles, exposed children; Open squares, comparison children. BRIEF®2, Behaviour Rating Inventory of Executive Function, 2nd edition; GEC, global executive composite; SDQ, Strength and Difficulties Questionnaire.

Considering the 27 children with both questionnaires completed satisfactorily (19 exposed and 7 comparison children), SDQ total difficulty scores and BRIEF®2 GEC scores were highly and positively correlated (*r* = 0.92, 95% CI 0.82–0.96, *p* < 0.0005, Figure 2). Treating classifications for each questionnaire as equivalent (SDQ high/very high ≡ BRIEF®2 potentially or clinically elevated; SDQ slightly elevated ≡ BRIEF®2 mildly elevated; SDQ close to average ≡ BRIEF®2 not elevated), 22/27 (81%) children had concordant classifications: 17 children were classified as normal on both, one child was mildly elevated/slightly raised on each, and four children were potentially or clinically elevated and high/very high on each (Figure 2). Of the five children with discordant classifications, one child had a BRIEF®2 score indicating more problems than their SDQ score, and four children had SDQ scores indicating more difficulties than their BRIEF®2 score.

### 3.4. Comparison with infant Griffiths scores, NAS/NOWS, and maternal methadone dose

Twenty-eight of these 33 children had attended for investigation at age of 6 months and had completed the Griffiths MD neurodevelopmental assessment (Table 2). Only 2 of these 28 had problematic infant scores: child #011 had a low eye-hand sub-quotient in infancy, but normal results on SDQ and BRIEF®2 at 8–10 years; child #018 had a low performance and eye-hand sub-quotients in infancy and also had normal SDQ and BRIEF®2 scores. Conversely, nine children with either an abnormal SDQ total difficulties score or an abnormal BRIEF®2 GEC at age 8–10 years had normal Griffiths scores in infancy.

Considering only exposed children, neither SDQ scores (subscales, total difficulties score) nor BRIEF®2 scores (subscales, indices, GEC) differed between children who did (*n *= 14) or did not (*n *= 7) require treatment for NAS/NOWS. Scatter plots of questionnaire scores and maternal methadone dose at delivery were random by visual inspection, indicating no relation between these two factors.

## 4. Discussion

The main aim of this study was to expand current literature on the developmental impact of prenatal opioid exposure for older children. We found that, at mid-elementary school age, children prenatally exposed to methadone and/or other drugs were not significantly different from their non-exposed peers in terms of carer reports on the SDQ screening questionnaire. On the BRIEF®2 measure, methadone-exposed children scored significantly higher than their non-exposed peer group on behaviour, emotional, and cognitive regulation indices as well as GEC, indicating that methadone-exposed children were significantly less able to cognitively regulate, control, and manage cognitive processes and problem solve in various contexts. After controlling for maternal tobacco use, however, methadone exposure was no longer a predictor of higher BRIEF®2 scores, with the BRI and CRI indices showing the largest adjustments. This may represent a type II error with this relatively small study as a large study of 92 methadone-exposed 2-year-old children and 108 unexposed control children found problems with motor function, cognitive development, and emotional/behaviour dysregulation persisted after controlling for confounding licit (including tobacco) and illicit drug use in pregnancy (9).

Prenatal tobacco exposure is known to be detrimental to brain development and function (14) with a long-term follow-up study linking tobacco exposure to reduced cognition (28). In a large US study of teenagers using teacher-reported BRIEF questionnaires, tobacco exposure was found to predict impaired behavioural regulation but not meta-cognition after controlling for multiple confounders including cocaine, alcohol, and cannabis (but not opioid exposure) (29). Our data support this finding by suggesting that tobacco exposure particularly exacerbates problems with behaviour regulation. Maternal smoking is significantly associated with childhood ADHD after adjusting for parental psychiatric history and socioeconomic status, but other confounders—such as opioid exposure—could not be included in the meta-analysis (30). It remains uncertain, therefore, whether tobacco and opioids act independently, exacerbate the other's teratogenic effect, or act as a marker for more extensive use. Studies investigating the safety of opioids in pregnancy must therefore control for maternal tobacco use (31).

Griffiths scores at 6 months were poorly predictive of 8–10 year outcomes. NAS/NOWS requiring treatment was not related to the presence or extent of any difficulties, suggesting that any prenatal exposure to opioids is a better risk factor for surveillance than a history of treated NAS/NOWS (1). The SDQ and BRIEF®2 tests correlated well across a wide range of scores, suggesting that non-significant SDQ findings may relate to the lower sensitivity of non-parametric testing used to compare SDQ scores between groups. The preponderance of children with difficulties highlighted by the SDQ but not by BRIEF®2 (*n* = 4) rather than vice versa (*n* = 1) is in keeping with the SDQ's design as a screening questionnaire.

Strengths of this study include being the first prospective cohort-based study describing longer-term neurodevelopmental effects of prenatal methadone exposure, uniquely comprehensive information on maternal substance misuse in pregnancy and a comparison group matched for gestation, birthweight, and postcode at delivery as a proxy for socioeconomic status. Limitations include the small sample size which reflects the difficulty of long-term follow-up of families with challenging and/or chaotic lives. The greater imprecision associated with small sample sizes may have masked any methadone effect after controlling for maternal tobacco use. Children in the exposed group had birthweights 236 g lighter on average and had smaller birth OFC by an average of 0.8 cm, in line with expected, corrected differences seen in methadone-exposed children (32), but not reaching significance likely due to the small sample size. A greater proportion of exposed children had poor vision: since vision tests were selected to be easily performed by younger children, visual findings are unlikely to be affected by behaviour or executive difficulties. It is possible that reported behaviour and/or executive difficulties were at least partly due to the presence of visual problems but because prenatal opioid exposure is associated with impaired vision (10), childhood vision outcome was not treated as a confounder. The comparison group had a higher proportion of females than the exposed group (6/8, 75%, vs. 9/19, 47%, with adequately completed BRIEF®2), which may have exaggerated positive findings in the exposed group as ADHD is more prevalent in males. Long-term outcomes for all children are confounded by multiple factors including impaired foetal growth, socioeconomic deprivation, and challenged parenting skills, each of which is more likely to affect those exposed prenatally to opioids, thereby limiting the strength of any association. However, multiple systematic analyses now point to an independent effect of prenatal opioid exposure on developmental outcomes (1, 6, 7, 10).

Opioid exposure *in utero*, specifically methadone, may at least partly explain adverse neurodevelopmental outcomes at mid-elementary school age, which are currently misunderstood or misdiagnosed, potentially as ASD or ADHD, by professionals. Unfortunately, potentially confounding effects of other illicit and licit drug exposures, challenged parenting and/or multiple placements (33), and socioeconomic deprivation are extremely difficult to control. Given national recommendations (4, 5) and the widespread use of methadone in the treatment of opioid use disorder in pregnancy, establishing whether this practice may contribute to long-term harm for children's developmental outcomes is essential.

## Data Availability

The raw data supporting the conclusions of this article will be made available by the authors, without undue reservation.
